# Are iodophor-impregnated drapes associated with lower intraoperative contamination compared to no adhesive drape?: A protocol for systematic review and meta analysis

**DOI:** 10.1097/MD.0000000000034641

**Published:** 2023-08-11

**Authors:** Albert González-Sagredo, Thiago Carnaval, Silvia Granados-Suárez, Robert Josua Cedeño Peralta, Paula López-García, Albert Castellà Durall, Sebastián Videla, Ramon Vila, Elena Iborra

**Affiliations:** a Angiology and Vascular Surgery Department, Bellvitge University Hospital, L’Hospitalet DE Llobregat, Barcelona, Spain; b Clinical Research Support Unit, Clinical Pharmacology Department, Bellvitge University Hospital, L’Hospitalet DE Llobregat, Barcelona, Spain; c Pharmacology Unit, Department of Pathology and Experimental Therapeutics, School of Medicine and Health Sciences, IDIBELL, University of Barcelona, L’Hospitalet DE Llobregat, Barcelona, Spain.

**Keywords:** iodine-impregnated drape, iodophor-impregnated drape, surgical drapes, surgical site infection, surgical wound infection

## Abstract

Surgical site infection (SSI) is one of the most frightening complications after surgery. Adhesive drapes (AD) are widely used as an infection prevention tool. They can be non-impregnated or iodophor-impregnated, although non-impregnated are less used as they might be related to higher number of infections. One of the most common ways to study their efficacy is by analyzing the intraoperative contamination, which is a useful primary endpoint as it does not need follow-up and it has been strongly associated with infections. Therefore, we believe a systematic review (SR) and meta-analysis is needed to determine which is the literature available about this topic and to explore their results.

**Methods:**

All randomized controlled trials (RCT) published since 1984 through to January 15, 2023 will be included. Non-human and experimental studies will be excluded. We will only include studies written in English. We will conduct searches in the following electronic databases: MEDLINE (via PubMed), SCOPUS and Web Of Science. The protocol of the SR was registered in PROSPERO under the number CRD42023391651 and was written according to the Preferred Reporting Items for Systematic Reviews and Meta-Analysis Protocol guidelines.

**Discussion:**

The evidence regarding the benefits of using iodophor-impregnated adhesive drapes (IIAD) is scarce. Therefore, this SR and meta-analysis is required to determine if they are related with a lower intraoperative contamination incidence, compared to no AD.

## 1. Introduction

Surgical site infection (SSI) is an infection that occurs after surgery in the part of the body where the surgery took place.^[1,2]^ For most SSIs, the source of the invading pathogen is the patient skin.^[3]^ Therefore, one of the commonly used strategies to reduce SSI is the use of adhesive drapes (AD), which act as a barrier to block the translocation of recolonizing bacteria from the skin adjacent to the surgical site into the surgical wound.^[4,5]^ They were first used in 1950 on abdominal surgery,^[6]^ and can be non-impregnated (NIAD) or iodophor-impregnated (IIAD), although NIAD are progressively being less utilized as some studies suggest that they might be associated with a higher SSI incidence.^[7–9]^ Consequently, the use of IIAD has increased in the past years, although the evidence on their role to prevent SSI is limited and only based on 2 randomized controlled trials (RCT).^[10,11]^ This scarce evidence is probably related to the fact that using SSI as a primary endpoint requires large samples and an extensive follow-up. Therefore, other primary endpoints such as intraoperative contamination have been increasingly studied.^[5,12,13]^ Surgical wound contamination has been established as a risk factor in the development of postoperative wound infection.^[14]^ Although a systematic review (SR) evaluating the effectiveness of IIAD on IC was conducted in 2021,^[15]^ it was only focused on orthopedic surgery and was restricted to 2 articles, limiting the validity of the meta-analysis. Furthermore, it did not include one of the referral studies on the topic, mentioned in the Cochrane review.^[8]^ Our SR and meta-analysis aims to include all the articles from all specialties published since the implementation of IIAD.

## 2. Methods

### 2.1. Study registration and ethics

The protocol of the SR was registered in PROSPERO (https://www.crd.york.ac.uk/prospero/) under the number CRD42023391651 and was written according to the Preferred Reporting Items for Systematic Reviews and Meta-Analysis Protocol guidelines.^[16]^

### 2.2. Type of studies

All RCTs published since 1984 through to January 15, 2023 will be included. Non-human and experimental studies will be excluded. We will only include RCTs written in English.

#### 2.2.1. Type of participants.

This study will include adult patients of any race undergoing clean surgery.

#### 2.2.2. Type of interventions.

The intervention group is those patients who received IIAD.

#### 2.2.3. Type of comparisons.

The control group is those patients in which no AD was used.

#### 2.2.4. Outcome measures.

The primary outcome will assess the percentage of contaminated swabs at the end of the surgery.

### 2.3. Data sources

We will conduct searches in the following electronic databases: MEDLINE (via PubMed), SCOPUS and Web Of Science. All bibliographic information and articles will be arranged using REDCap.

### 2.4. Selection of studies

Two independent reviewers (AG-S and TC) will select studies according to the process of the SR. First, the reviewers screen the tittles and abstracts to select relevant studies. They will then double check by reading the full text and remove duplicate publications and non-relevant studies. Disagreements will be resolved by discussion or consultation with the third reviewer (SV). Details of the selection process will be summarized in a PRISMA 2020 flow diagram.^[17]^

### 2.5. Data extraction

We will prepare an Excel sheet (Microsoft Office) for data extraction. Two reviewers will independently extract data from the selected studies and import them into the form. All disagreements will be resolved through discussion with a third author. The following data will be included: the first author, year of publication, interventions, and comparison treatment, duration, follow-up, outcome measurements, results, and other detailed information.

### 2.6. Quality assessment

Two reviewers will independently evaluate the included studies by using the Cochrane Handbook for Systematic Reviews of Interventions (version 6.3) as a guide. There are 7 aspects to the evaluation: random sequence generation, allocation concealment, blinding of participants and personnel, blinding of outcome assessment, incomplete outcome data, selective reporting bias, and other biases.^[18]^ Every item will be classified as low, high, or unclear. Unresolved problems are resolved by a third reviewer (SV).

### 2.7. Data synthesis

Statistical analyses will be conducted using R version 4.1.0 por Windows [R Core Team (2020). R: A language and environment for statistical computing. R Foundation for Statistical Computing, Vienna, Austria. URL: https://www.Rproject.org/]. The differences between the intervention and control groups will be assessed in this study. For continuous data, we use the mean difference and standard deviation with a 95% confidence interval (95%CI) to measure the treatment effects. For outcome variables on different units, we use the standard mean difference with 95%CI. For dichotomous data, we will use treatment relative risk with 95%CI to assess the treatment effect. The analysis will use a random or fixed model according to the type of data. If quantitative synthesis is not appropriate, we will summarize the findings of studies and draw a conclusion. We will use the Mantel-Haenszel method to calculate the common effect estimate, using its random variant in the random case to account for inter-study heterogeneity, and applied the truncated Knapp-Hartung adjustment to the standard error to provide conservative confidence limits with enhanced coverage.

### 2.8. Unit of analysis issue

For crossover studies, we use data from the first treatment period. If the clinical trials are assessed in more than one control group, we will implement the primary analysis to combine the data from each control group. Subgroup analyses of the control groups will also be conducted. Each patient will be evaluated only once during the analyses.

### 2.9. Dealing with missing data

If data of the study are missing or incomplete, we will contact the corresponding authors by email. However, if it is not possible or there is no reply from the author, the study will be excluded.

### 2.10. Assessment of heterogeneity

If the included clinical trials can be implemented in a meta-analysis, the *I^2^* tests will be exploited to appreciate the heterogeneity of studies, where *I^2^* > 50 will indicate high heterogeneity. In the case of heterogeneity, subgroup analyses will be performed to explore the possible reasons.

### 2.11. Sensitivity analysis

If there is adequate sample size, high-quality methodology, and low heterogeneity, sensitivity analysis will be performed to assess the robustness of the study.

### 2.12. Ethics

Ethical approval was not necessary as no individual data was used.

### 2.13. Assessment of reporting biases

If the final selected studies are of sufficient quantity (at least 3 trials), we will use funnel plots to detect whether reporting bias exists.

## 3. Discussion

SSI is one of the most frightening complications in vascular surgery.^[19–22]^ The rational for this SR is that the use of AD could have an impact on SSI incidence. NIAD has been demonstrated to not only not reduce SSI incidence, but to increase it compared to bare skin,^[7–9]^ therefore NIAD will not be included in this SR. We will focus on the comparison between IIAD and bare skin, as there is not enough evidence to stablish a strong recommendation on which of the 2 methods is better in terms of reducing SSI.^[8,10,11]^ There is scarce evidence regarding the association between IIADs and SSI. Therefore, we will use as a primary endpoint the IO as before studying the effect on SSI we must be aware if it firstly reduces the surgical site contamination.^[5,12,13]^ If affirmatively reduces intraoperative contamination, then we will perform a RCT comparing both techniques to assess which is associated with a lower SSI incidence. This SR will search various databases and provide comprehensive evidence for several recommendations that may be advantageous for patients, researchers, and policymakers in the clinical environment.

## Acknowledgments

We would like to thank the IDIBELL Foundation and the CERCA Program/Generalitat de Catalunya for the institutional support provided.

## Author contributions

**Conceptualization:** Albert González-Sagredo.

**Data curation:** Albert González-Sagredo, Thiago Carnaval.

**Formal analysis:** Albert González-Sagredo, Thiago Carnaval.

**Funding acquisition:** Albert González-Sagredo.

**Investigation:** Albert González-Sagredo, Thiago Carnaval.

**Methodology:** Albert González-Sagredo, Thiago Carnaval.

**Project administration:** Albert González-Sagredo.

**Resources:** Albert González-Sagredo.

**Software:** Albert González-Sagredo.

**Supervision:** Albert González-Sagredo, Sebastián Videla, Ramon Vila, Elena Iborra.

**Validation:** Albert González-Sagredo, Silvia Granados-Suárez, Robert Josua Cedeño Peralta, Paula López-García, Albert Castellà Durall, Sebastián Videla, Ramon Vila, Elena Iborra.

**Visualization:** Albert González-Sagredo.

**Writing – original draft:** Albert González-Sagredo.

**Writing – review & editing:** Albert González-Sagredo, Sebastián Videla, Ramon Vila, Elena Iborra.
